# Regional Brain Changes Occurring during Disobedience to “Experts” in Financial Decision-Making

**DOI:** 10.1371/journal.pone.0087321

**Published:** 2014-01-24

**Authors:** Victoria Y. M. Suen, Matthew R. G. Brown, Randall K. Morck, Peter H. Silverstone

**Affiliations:** 1 Department of Psychiatry, Faculty of Medicine and Dentistry, University of Alberta, Edmonton, Canada; 2 Department of Finance and Statistical Analysis, Faculty of Business, University of Alberta, Edmonton, Canada; Middlesex University London, United Kingdom

## Abstract

It is well recognized that individuals follow “Expert” advice, even when flawed and offers no advantage, and sometimes leads to disadvantages. The neurobiology underlying this is uncertain, and in particular there is an incomplete understanding of which brain regions are most involved when individuals chose to disobey an expert. To study this we examined functional magnetic resonance imaging (fMRI) differences during an investment game where subjects received differentially credible investment advice. Participants (n = 42; 32 males) played an investment game, in which they could Buy or Not Buy a sequence of stocks. The better they did, the more money they made. Participants received either “Expert” advice or “Peer” advice. Those receiving Expert advice were told the advice came from a certified financial “Expert”. Those receiving Peer Advice were told the advice was that of the student administering the scans, who deliberately dressed and acted casually. Both streams of advice were predetermined and identical. The advice was scripted to be helpful initially, but progressively worse as the task continued, becoming 100% wrong by the end of the task. Subjects receiving Expert Advice followed the advice significantly longer on average, even though this was progressively worse advice. Thus, following Expert advice had poorer consequences for individuals, but this did not dissuade them from continuing to follow the advice. In contrast, when subjects disobeyed Expert advice they exhibited significant anterior cingulate cortex (ACC) and superior frontal gyrus activation relative to those disobeying Peer advice. These findings may suggest that in subjects who defy authority, or believe they are doing so (in this case by disobeying an “Expert”) there is increased activation of these two brain regions. This may have relevance to several areas of behavior, and the potential role of these two brain regions in regard to disobedience behavior requires further study.

## Introduction

In this study we find that when individuals defy authority, or believe they are doing this (in this case by disobeying an “Expert”) to make an investment decision there is increased activation of the anterior cingulate cortex and the superior frontal gyrus, and it is conceivable that this activation may, in part, be responsible for disobedience. This may have relevance to several areas of behavior. Certainly, it has long been known that individuals follow “expert” advice, even when it is of dubious origin [1], [2], [3] and this also occurs when individuals seek financial advice [4]. Professional money managers’ substantial underperformance net of fees [5] is estimated to cost investors almost two-thirds of a percent per year [6], [7]. Including other fees they pay to the brokers and investment advisors who direct them to underperforming money managers’ mutual funds [8], [9] raises investors’ losses to 2% per year relative to index funds. Compounded over the years until the typical investors’ retirement, this constitutes a huge transfer of wealth [10]. However, the brain regions involved in decision-making when individuals choose to follow, or not follow, “expert” advice remains uncertain, although expert advice is valued higher than novice advice and this choice does affect specific brain regions [11].

**Figure 1:**
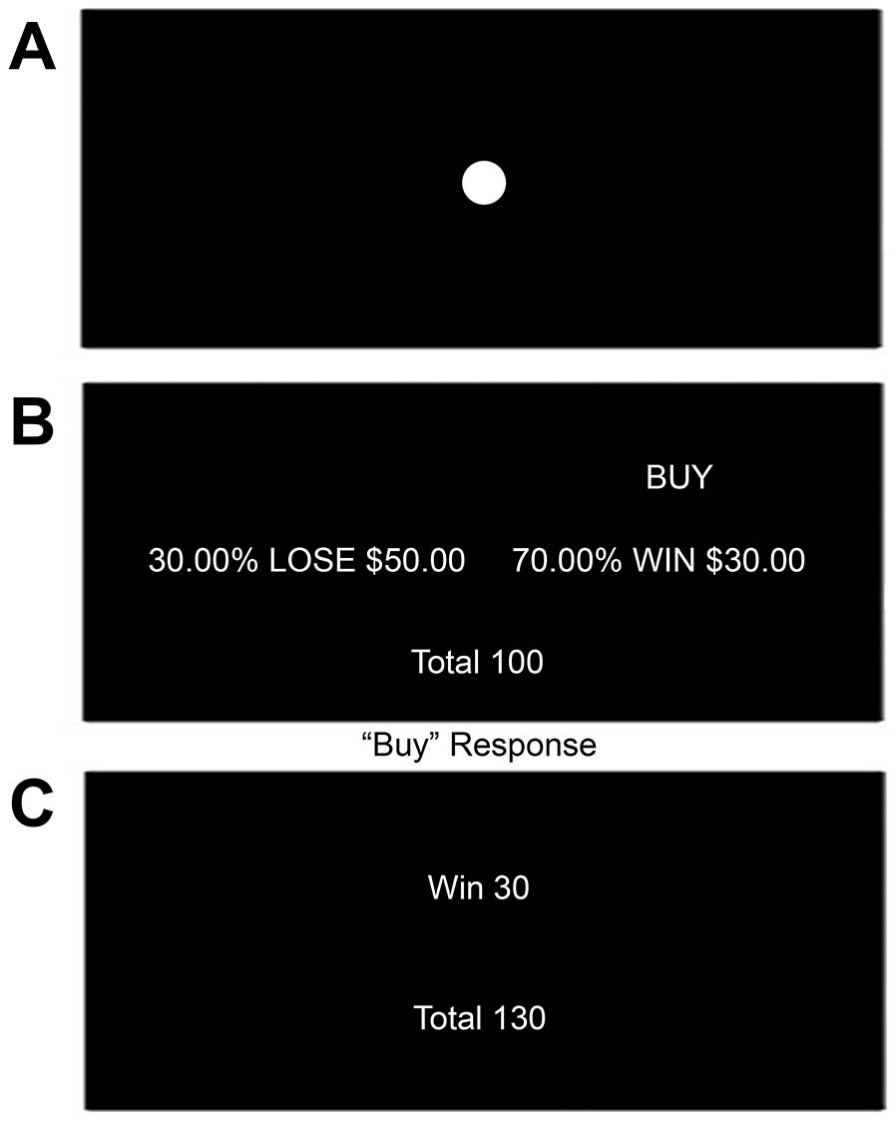
Investment Task. **a:** Fixation Point (6–10s): Participants were instructed to attend to the fixation point **b:** Trial (7s): Participants are presented with a stock and must decide to either “Buy” or “Not Buy”. Advice to “Buy” is rational as the expected value of buying the stock (0.7×30 = 21) outweighs the expected value of not buying the stock (0.3×–50 = –15). **c:** Feedback (1s): Participants are presented with feedback based on their decision (in this case the participant chose to obey the advice and “Buy” thus the trial resulted in a win) and their total is adjusted accordingly.

To further explore these issues, we utilized an experimental design in which subjects make a series of investment decisions, with some receiving what they believe to be “Expert” Advice and others receiving “Peer” Advice (which is in fact identical to the “Expert” Advice). The advice was initially correct, but grew increasingly useless over time. Using functional magnetic resonance imaging (fMRI) we determined which brain regions activated when individuals choose to obey or disobey an “Expert”, paying particular attention to changes around the time that individuals chose to “disobey” a discredited expert. We asked participants to choose to “Buy” or “Not Buy” stocks in a simple stock-trading game (Figure 1). The better they did during the task, the more money they accumulated. Subjects actual earnings varied from $45 to $105, so their potential earnings created a credible incentive to try and do well.

Trials were presented either with or without advice. Participants were divided into two groups. When advice was given about whether to “Buy” or “Not Buy” one group was told that the advice came from an experienced financial “Expert” with over 20-years of financial experience, while the other was told the advice was from the PhD student conducting the study (“Peer”), although when advice was presented this was always identical for every answer for both groups. The advice deteriorated from being 100% correct at the beginning of the study session (always accurately reflecting the expected value of the stock’s payoffs), to being 100% wrong by the end of the session (always inaccurately reflecting the expected value of the stock’s payoffs; Table 1). By following the advice at the beginning parts of the study the subjects would always increase their winnings, but by the end of the study if they followed the advice they would always decrease their winnings (and therefore would take less money home). We were interested in changes in brain activation that occurred when participants disobeyed the advice.

**Table 1:** Investment task conditions.

Trials	Type of Advice	Type of Buy	Number of Trials
First 1/3 of trials Runs 1 and 2	No Advice	Good Buy	8
		Bad Buy	6
	Good Advice	Good Buy	12
		Bad Buy	12
**Second 1/3 of trials Runs 3 and 4**	Good Advice	Good Buy	18
		Bad Buy	16
	Bad Advice	Good Buy	16
		Bad Buy	16
**Last 1/3 of trials Runs 5 and 6**	No Advice	Good Buy	8
		Bad Buy	6
	Bad Advice	Good Buy	12
		Bad Buy	12

We expected to see brain activation consistent with previous studies of decision-making under conditions of uncertainty. Previous research in risky decision-making has implicated a wide distribution of areas including the ventromedial prefrontal cortex, insular cortex, parietal and temporal cortices as well as areas of the striatum [12], [13], [14]. We expected our task would elicit similar patterns of activation at its most basic decision level: choosing to “Buy” or “Not Buy” a stock.

We also anticipated several advice related differences between groups. First, if following expert advice is a cognitive shortcut that minimizes costly “slow thinking” by offloading onto an “Expert”, individuals who follow “Expert” advice were expected to exhibit minimal brain activation. Previous research has shown that expert advice can significantly alter decision-making both behaviorally and neurobiologically [15]. We expected to see a decrease in cognitive effort yielding less activation when advice was presented.

Secondly, if obeying an expert counters anxiety and evokes good feelings associated with “trusting an expert”, evidence of less anxiety and/or positive emotions might also be detectable in individuals who follow expert advice. Activation in the ventral striatum, orbitofrontal cortex, ventrolateral prefrontal cortex and anterior insula have been associated with processing positive emotions and rewards [16], [17], [18] and were expected to support the hypothesis that following expert advice elevates an investor’s utility.

Thirdly, we expected that either individuals receiving “Peer” advice should exhibit less advice-related activation compared to those receiving “Expert” advice or individuals receiving “Peer” advice should be less influenced by that advice and exhibit greater brain activation than those receiving “Expert” advice. Advice provided by a “Peer”, if less influential, should lead to decreased activation in the advice related areas activated in individuals provided with “Expert” advice. Moreover, “Peer” advice could lead to more activation associated with problem solving in subjects who deem this advice less valuable than advice from an “Expert”.

Lastly, we were particularly interested in the activation elicited when the proposed obedience reflex was disengaged, or more simply when individuals chose to disobey the presented advice. Individuals given financial advice by an “Expert” contrary to their financial interest were expected to exhibit greater response conflict, potentially related to disengaging an obedience reflex, and perhaps stronger negative emotions, when they chose to disregard that advice than would individuals given advice by a “Peer”. Negative emotions and punishment have been associated with activation in the amygdala, orbitofrontal cortex, ventrolateral prefrontal cortex and anterior insula [18], [19] and such activation was expected in decisions to disobey the “Expert” advice. We anticipated differences in brain activation related to response conflict in previously identified brain regions of interest. Certain brain regions are activated in studies of decision-making, including the anterior cingulate cortex (ACC), anterior and posterior lateral prefrontal cortices, medial frontal cortex, insular cortex, intraparietal sulcus, striatum, and thalamus [11], [15], [20]. Previous studies using a variety of paradigms with both animal and human subjects support a central role for the prefrontal cortex in decision-making. The ACC is implicated in particularly complex decisions involving ambiguity, conflict and increased potential for errors [21], [22], [23], [24]. Therefore, these regions might be involved when individuals choose to “obey” or “disobey” an “Expert”.

To our knowledge, this is the first fMRI study to employ a task simulating investment decision-making based on advice that is manipulated in terms of both the expertise of the individual giving the advice as well as the accuracy of the advice throughout the task.

## Materials and Methods

### Ethics statement

This study was approved by the Ethics review board of the University of Alberta. All participants signed an informed consent form. The following data will be freely available to those who contact the corresponding author.

### Recruitment and Study Process

Participants were recruited from the University of Alberta and surrounding Edmonton area. All potential participants received a brief summary of the study. Individuals were screened for the presence of any psychiatric disorder, using a semi-structured interview, as well as for any risk associated with having an MRI scan. Following this screening process, a total of 48 individuals (32 males, age range 20 – 39) were eligible and willing to take part in this study.

### Investment paradigm completed by individuals

Participants completed an investment task during their fMRI scan. Participants were told that the investigators were interested in how individuals make investment decisions. On each trial, participants were asked to decide to either ‘Buy’ or ‘Not Buy’ a stock based on the following information: the probability of winning a specified amount of money, the probability of losing a specified amount of money, and advice on what action to take. An example of the sequential images shown to each individual per investment decision is shown in Figure 1. Each investment choice was presented for seven seconds and participants were instructed to make their decision within that time frame. All participants were told that advice was being presented to aid in their decision-making process but that they were not required to follow it if they did not want to.

Each participant began with a nominal total of $100. If they ended the task with the same amount (or less) in nominal dollars they would take home $45. However, if they increased their earnings in the task, they would be given greater compensation, with a take home amount ranging between $45 – $105. By using real financial incentives participants were more likely to increase the amount of cognitive effort put into the task [25] and act in ways that more closely mimic real world investing decisions [25], [26].

The outcome of each trial was pre-determined based on the expected value of the stock presented (i.e. the probability of either the win or loss multiplied by the dollar amount associated with that probability). Thus, a participant would win the trial if he/she chose to buy the stock and the expected value of the probability of winning money on the stock was greater than that of losing money. For example, if the stock presented had a 70% chance to win $50 and a 30% chance to lose $100, the expected value of winning is 35 (0.7×50) while the expected value of losing is −30 (0.3×−100). As the expected value of winning outweighs the expected value of losing (the addition of 35 and –30 yields a positive number), the rational decision would be to buy this stock. If participants chose to buy a stock whose expected value for a loss outweighed that for a win, that decision would result in a loss on that trial. For example, if the stock presented had a 40% chance to win $90 and a 60% chance to lose $80, the expected value of winning is 36 (0.4×90) and the expected value of losing is −48 (0.6×−80). As the addition of 36 and –48 yields a negative number, in this case the rational decision would be to not buy the stock. It was possible in theory for a participant to complete the task in an entirely rational manner by simply calculating the expected value for every stock and choosing the appropriate action.

In terms of how the lotteries were constructed – 10/90%, 20/80%, 30/70%, 40/60% probability splits were used. Lotteries were assigned low medium and high difficulty based on how great a difference there was in expected values - smaller difference in expected values were more difficult trials than ones with large differences in expected values. The presentation of trials was counterbalanced with regard to advice and difficulty in order to control for order effects.

Trials were followed by one second of feedback, as follows:

- If a participant chose to buy the stock they were informed if that choice resulted with a win or a loss, and their total was adjusted accordingly. Each win and loss amount was determined by the stock presented.
- If a participant chose not to buy the stock the feedback read “No Buy”, and the total remained the same.
- If a participant failed to respond in the allotted time, the feedback read “No Response”, and the total remained the same.

After completion of the task in the scanner, participants were asked what strategies they used to make their decisions and also for feedback on the advice that was given to them.

Nonetheless, we recognize that the design was somewhat complex, although we were constrained by some technological issues in the paradigm, for example the speed of response in decision-making versus what is detectable utilizing fMRI. For the same reasons we didn’t utilize a simpler task, as although we recognize that calculation of expected values is difficult for many individuals, particularly in the time required, it did allow us to clearly study our key variable. Thus, we were most interested in how closely individuals followed “Expert” advice, even when the utility of this was clearly decreasing over time.

### Information given to participants regarding the advice they received

Participants were divided into two groups based upon what they were told regarding the advice they received about investment decisions during the investment paradigm. Advice as to whether to “Buy” or “Don’t Buy” appeared in 80% of the trials (Table 1). Participants in the external expert group (“Expert”) were instructed that the advice was given by an outside financial expert with over 20 years of experience in the field of financial investments who had been specifically asked to prepare the advice he would give his own clients in such a situation. Participants in the peer group (“Peer”) were instructed that the student who was in charge of running the study was giving the advice. This was a single-blind study in which participants were randomly placed into alternating blocks of either the “Expert” (28 participants) or “Peer” (20 participants) groups. All other aspects of information given to the subjects were identical, as was the training they received. All participants were aware of the order of each task stimulus, what actions they could take and what the consequences of those actions could be. Each participant was allowed a practice run of the task that was equivalent in length to the first run that they would complete in the MRI scanner. During the practice run of 19 trials, seven were “No Advice” trials while the remaining 12 were all “Good Advice” trials.

Although participants were not aware of this, all advice was pre-determined and did not differ between groups. The schedule was designed to create credibility for the advice, which would gradually become less and less rational. Thus, the paradigm was divided into six runs. During the first 2 runs, if the suggested advice was followed it would result in a win or no loss (Good Advice). During runs 3 and 4, the advice gradually became less reliable (50:50 mixture of Good Advice and Bad Advice). During runs 5 and 6, whenever the advice was followed this would result in a loss or a failure to win (Bad Advice). Advice was not given in every trial. Thus, as the advice became increasingly questionable, participants were required to choose between obeying external advice or not, with the only difference between the groups being whether or not they believed it came from an “Expert” or “Peer”. The number of times the various choices (good advice, bad advice, and no advice) was given is shown in Table 1.

### fMRI acquisition

A 1.5-T Siemens scanner and 8-channel head coil was used for data acquisition at the University of Alberta’s Peter S Allen MR Research Centre. Thirty-two axial slices (3×3×4 mm voxels) were acquired in a descending interleaved order. Functional images were obtained using a gradient echo EPI sequence (TR  =  2000 ms, TE  =  40 ms, FOV  =  256 mm, flip angle  =  90°). One hundred forty-four slices were acquired with a T1-weighted pulse sequence in the same location for structural images (MPRAGE, TR  =  1670 ms, TE  =  3.82 ms, TI  =  1100 ms, flip angle – 15°, FOV  =  256, 1 mm thick). Images were pre-processed and analyzed using SPM8. Pre-processing steps included 6-parameter rigid body motion correction, slice timing correction, and co-registration to each participants’ anatomical image to their functional scans. Structural scans were normalized to the Montreal Neurological Institute (MNI) template, and functional images were normalized to the new anatomical image. Lastly, we performed smoothing using a three-dimensional Gaussian filter (8-mm FWHM). Five participants (four from the “Expert” group; one from the “Peer” group) were excluded from further analyses due to significant movement artifacts that occurred during the scans (pitch, roll or yaw translation greater than 8 mm).

### Statistical Analysis

Behavioral data on the investment task was analyzed using SPSS 21. A 2×3 ANOVA and independent samples two-tailed *t*-test were performed to determine if obedience was mediated by condition.

fMRI data were analyzed using the General Linear Model. During model specification, trials were classified by type of advice (No Advice, Good Advice, Bad Advice), type of buy (Good Buy resulting in a win, Bad Buy resulting in a loss), and decision (Buy, Not Buy). Nuisance predictors included run offsets and six motion parameters. We included the trials from all runs in a single General Linear Model, grouping together run 1 with run 2, run 3 with run 4, and run 5 with run 6, as per the type of advice provided. GLM parameters were estimated using linear least-squares error fitting. We computed the following first-level statistical contrasts separately for each participant: Buy – Did Not Buy, Did Not Buy – Buy, Advice – No Advice, No Advice – Advice, Obedient – Not Obedient, Not Obedient – Obedient (Obedient and Not Obedient trials, respectively, were defined as those in which the participant's choice matched / did not match the advice), Good Advice – Bad Advice, and Bad Advice – Good Advice. We performed three second level analyses on the amplitudes of each contrast: within group t-test across all participants in the "Peer" group to detect significant contrast amplitude, within-group t-test across all participants in the "Expert" group, and between-groups t-test comparison. For all analysis, we used a voxelwise statistical threshold of *t*(40)  = 2.0211 (*p*<0.05 uncorrected) and a cluster size threshold of k = 201 voxels, yielding *p*<0.05 corrected for multiple comparisons across both the voxel population as well as the statistical tests. Cluster size threshold level was computed using Monte Carlo simulation.

To examine the effect of obedience on brain activity over time we conducted a 2 (Group; “Expert” vs. “Peer” Advice) x 2 (Run; Obedient versus Not Obedient runs 1 and 2 vs. Obedient versus Not Obedient runs 5 and 6) ANOVA. Four participants (three from the “Expert” group and one from the “Peer” group) were excluded from this analysis due to being 100% obedient in the first two runs thus Obedient versus Not Obedient runs 1 and 2 contrasts could not be computed. To elucidate what was driving the interaction effect, four two sample t-tests were conducted and each used as a mask for the interaction contrast.

## Results

### Behavioral Results

Post-scan responses were collected to ensure that participants believed that either an outside financial “Expert” or the student running the experiment (“Peer”) was giving the advice. No participants indicated suspicion of the indicated advisor. One participant (“Expert” group) indicated only following advice and not attempting to form independent judgments for each stock and was subsequently excluded from all analysis. The remaining 42 participants acknowledged use of the advice as an aid in decision-making at the beginning of the task.

**Figure 2:**
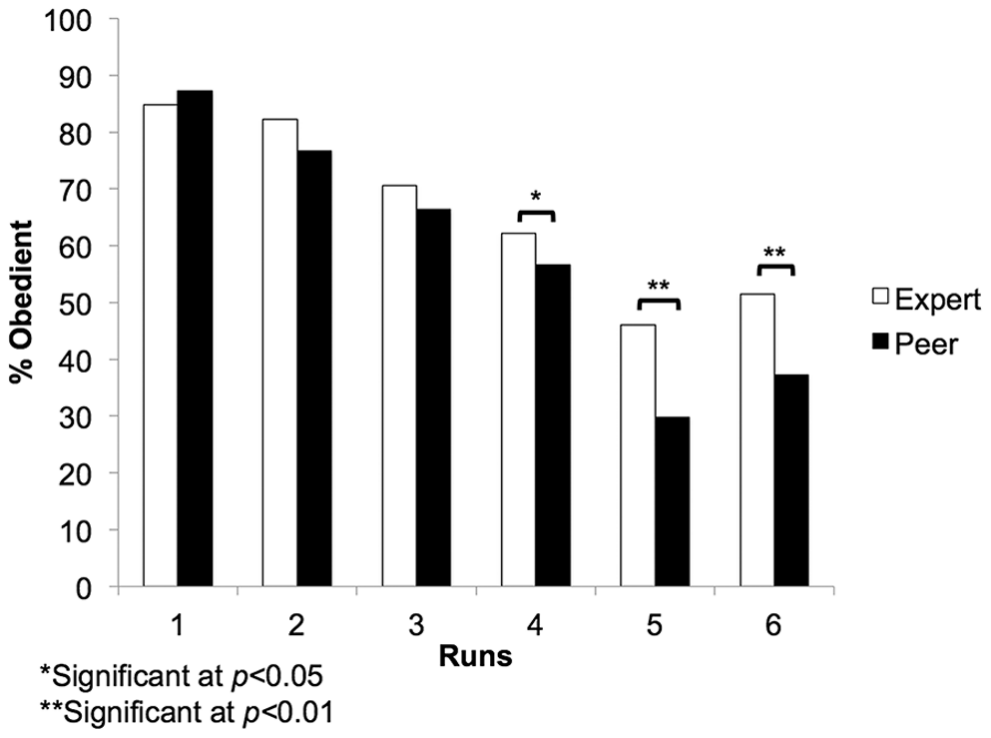
Comparison between groups in obedient decisions. Significant differences in number of obedient decisions in the last half of the study session (runs 4–6): Run 4(*t*(40)  = 2.08, *p* = 0.044), Run 5 *(t*(40)  = 3.47, *p* = 0.001) and Run 6 *(t*(40) = 3.09, *p* = 0.004). The “Expert” group was significantly more obedient to the advice in the last three runs than the “Peer” group.

However, in both groups all participants indicated using personal strategies as their main decision tool, citing an increased comfort with the task and a lack of trust in the advisor as the task continued. Interestingly, rates of obedience differed significantly between the two groups (*t*(40)  = 2.48, *p* = 0.018), with the “Expert” group (obedient on 30.38 ± 4.29% of trials with advice) obeying the advice significantly more often than the “Peer” group (27.44 ± 3.19%). As the study session progressed, participants exhibited decreasing rates of obedience to the advice as it went from good to mixed to bad. This effect was larger in the “Peer” group than the “Expert” group (Figure 2). We conducted a 2-way ANOVA and found significant main effect for Group (“Expert” vs. “Peer”; *F*(1,1240)  = 17.951, *p*<0.0001) and Run (1–6; *F*(5,240)  = 84.126, *p*<0.0001). The interaction effect was also significant (*F*(5,240)  = 2.744, *p* = 0.020).

### Neuroimaging Results

**Figure 3:**
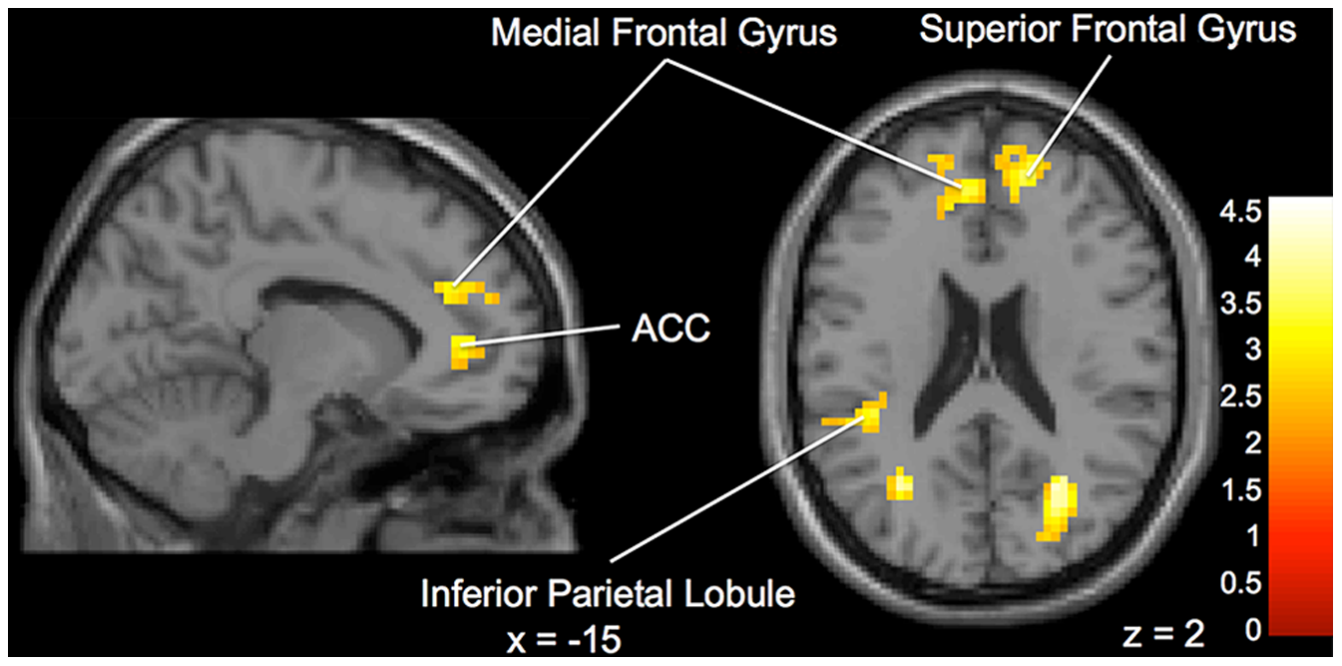
Brain activation for statistical contrast maps *NOT OBEDIENT – OBEDIENT*. “Expert” group shows increased activation compared to “Peer” group in the ACC, medial frontal gyrus and superior frontal gyrus. “z” and “x” coordinate provided at bottom right corners in MNI space. All results voxelwise statistical threshold at t  =  2.0211 (p < 0.05) and a cluster threshold level of k  =  201, p < 0.05 corrected for multiple comparisons.

**Figure 4:**
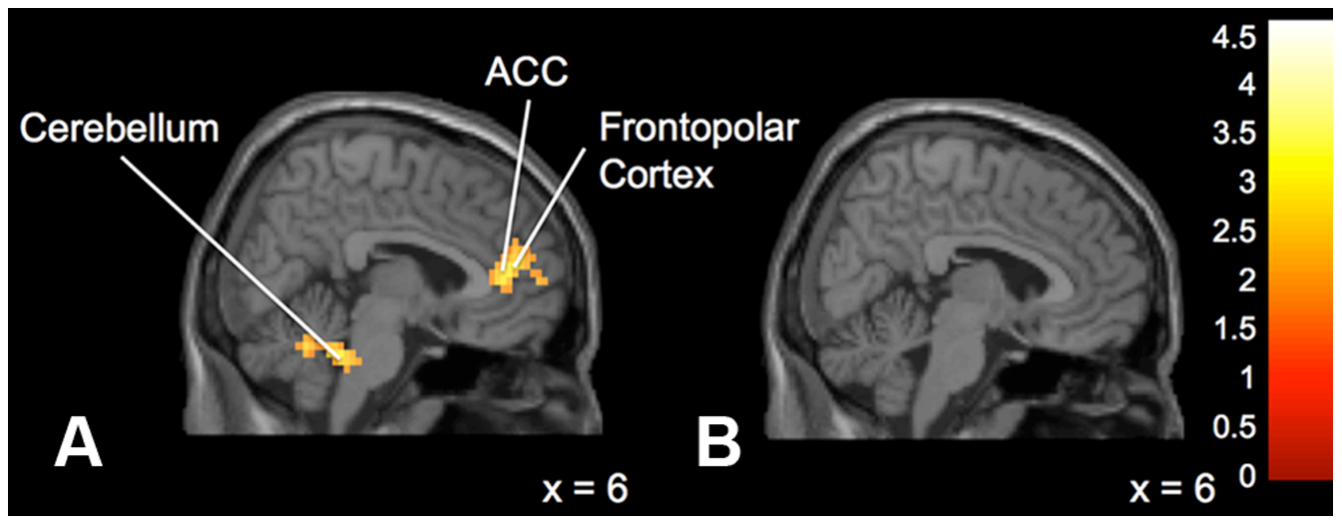
Brain activation for statistical contrast maps *NOT OBEDIENT – OBEDIENT* (within-groups). **a:** “Expert” group. Not Obedient trials elicited activation in the left anterior cingulate cortex, frontopolar cortex and cerebellum in the “Expert” group **b:** “Peer” group. No significant activation was found in Not Obedient trials in the “Peer” group. “z” coordinate provided at bottom right corners in MNI space. All results voxelwise statistical threshold at *t* = 2.500 (*p*<0.01) and a cluster threshold level of *p*<0.05 corrected for multiple comparisons.

**Figure 5:**
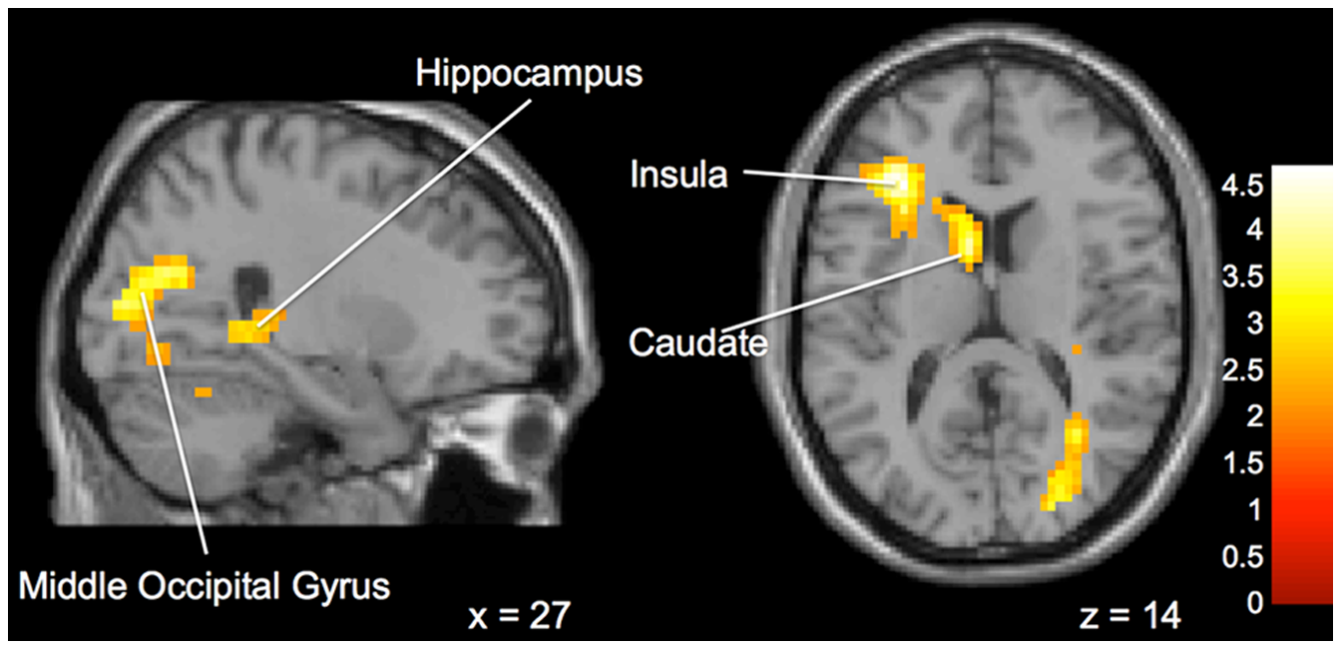
Brain activation for statistical contrast maps *OBEDIENT – NOT OBEDIENT*. “Peer” group shows increased activation compared to “Expert” group in the middle occipital gyrus, hippocampus, insula and caudate. “z” and “x” coordinate provided at bottom right corners in MNI space. All results voxelwise statistical threshold at *t* = 2.0211 (*p*<0.05) and a cluster threshold level of k = 201, *p*<0.05 corrected for multiple comparisons.

**Obedient versus Not Obedient.** Differences emerged between the “Expert” and “Peer” groups when choosing to follow or not follow the advice presented. The “Expert” group displayed significantly greater activation in left anterior cingulate cortex, right superior frontal gyrus, left inferior parietal lobule, left medial frontal gyrus, and left frontal lobe and bilateral temporal lobe white matter (Figure 3) during Not Obedient (or “disobedient”) trials (when compared to Obedient trials). On within group tests, when participants in the “Expert” group disobeyed the advice, there was significant activation in bilateral anterior cingulate, right frontopolar cortex, the right pons and left culmen (Figure 4). In contrast, in the same comparisons the “Peer” group displayed more activation in the right temporal lobe, left insula, right middle occipital gyrus, right hippocampus and left caudate (Figure 5).

**Figure 6:**
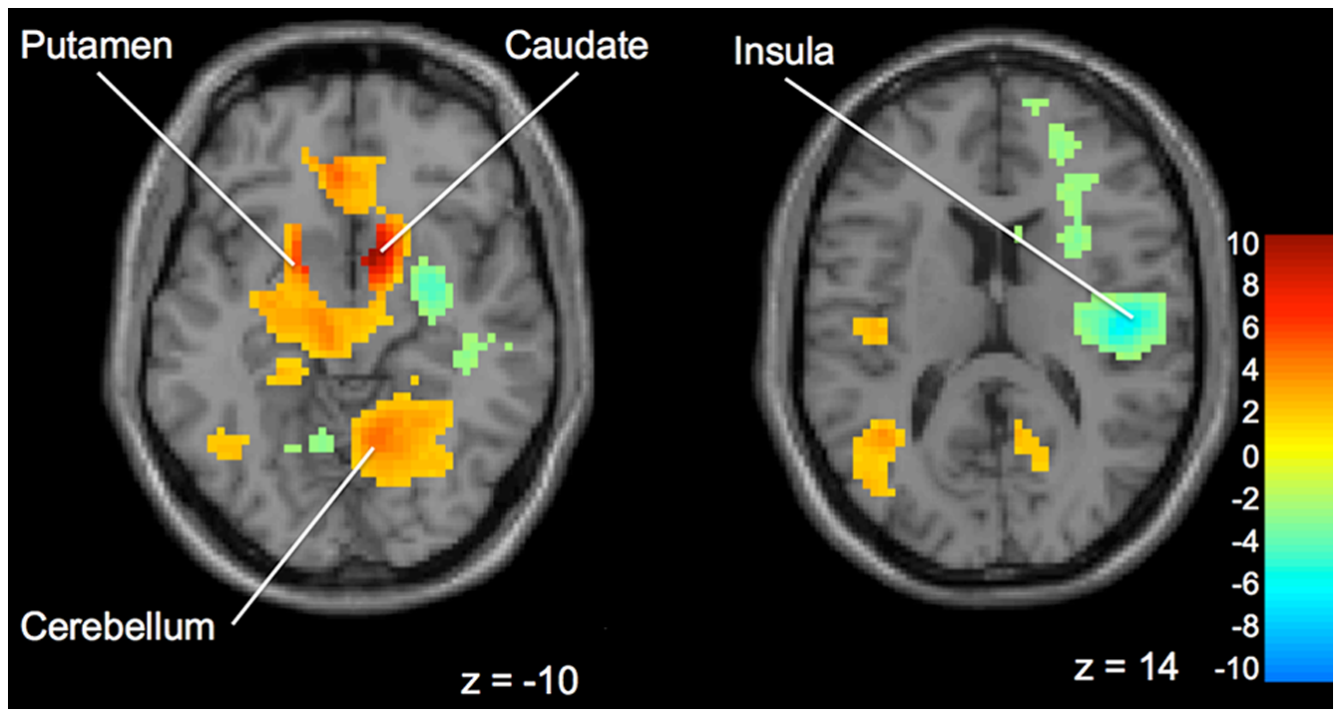
Brain activation for statistical contrast maps *BUY – DID NOT BUY* and *DID NOT BUY – BUY*. **a:** Brain activation for statistical contrast maps *BUY – DID NOT BUY* **.** Buy trials included a significant cluster of activation in the right caudate and cerebellum in both groups. **b:** Did Not Buy trials activated the right insula in both groups. “z” coordinate provided at bottom right corners in MNI space. All results voxelwise statistical threshold at *t* = 2.0211 (*p*<0.05) and a cluster threshold level of k = 201, *p*<0.05 corrected for multiple comparisons.

**“Buy” versus “Not Buy”.** No significant differences were found between the “Expert” and “Peer” groups when a decision to “Buy” or “Not Buy” a stock was made. In both the “Expert” and “Peer” groups, there was increased activation in the striatum (specifically the right caudate and left putamen), left pallidum, left middle temporal gyrus, and right cerebellum when participants chose to “Buy” (Figure 6a). In contrast, when participants chose to “Not Buy”, there was significant activation in the right insula, left cerebellum, cingulate gyrus, right middle frontal gyrus and right inferior parietal lobule (Figure 6b).

**Figure 7:**
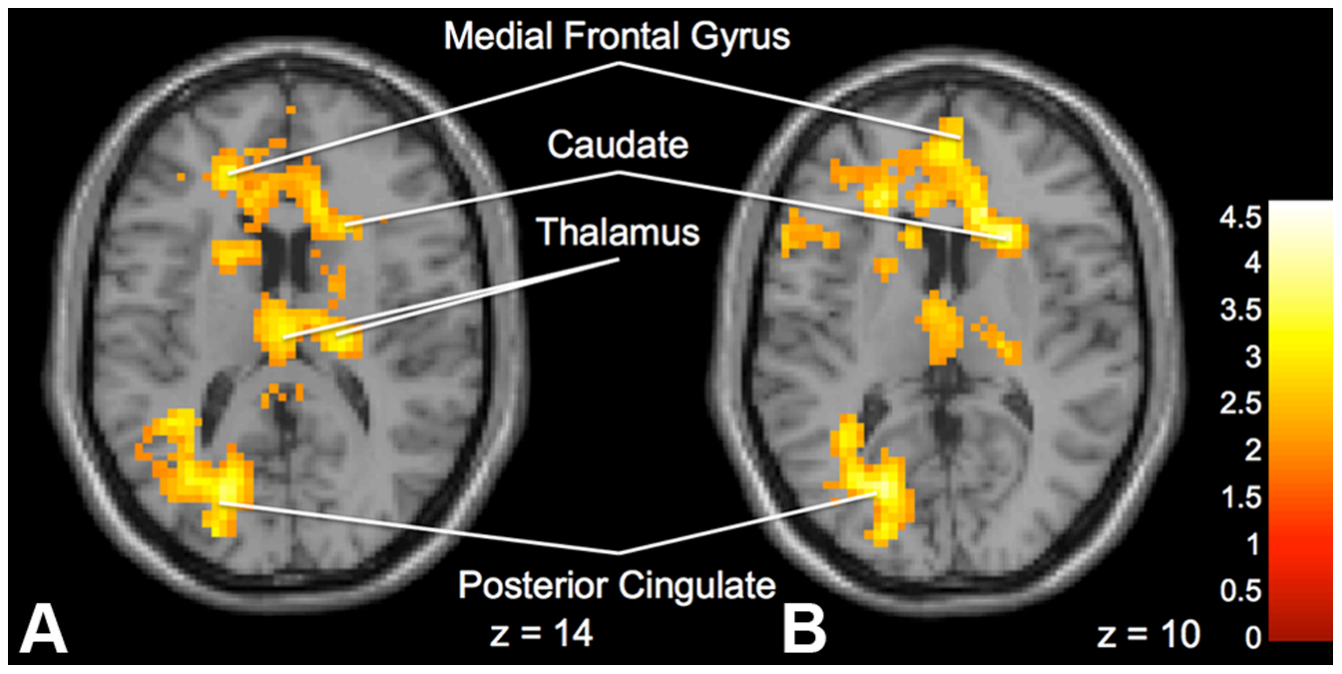
Brain activation for statistical contrast maps *ADVICE – NO ADVICE* and *NO ADVICE – ADVICE*. **a.** “Peer” group shows increased activation compared to “Expert” group in the posterior cingulate, medial frontal gyrus and caudate in the *ADVICE – NO ADVICE* comparison. **b.** “Expert” group shows increased activation compared to “Peer” group in the posterior cingulate, medial frontal gyrus, caudate and thalamus in the *NO ADVICE – ADVICE* comparison. “z” coordinate provided at bottom right corners in MNI space. All results voxelwise statistical threshold at *t* = 2.0211 (*p*<0.05) and a cluster threshold level of k = 201, *p*<0.05 corrected for multiple comparisons.

**Advice versus No Advice.** When comparing Advice to No Advice trials, there was significantly greater activation in the “Peer” group compared to the “Expert” group in the left posterior cingulate cortex, right caudate, left insula, right medial frontal gyrus, left middle frontal gyrus and bilateral frontal lobe white matter (Figure 7a). On within group tests, no significant differences were found in the “Expert” group. However, significant activation emerged in the “Peer” group in the right temporal middle gyrus, left calcarine, left cerebellum, left lingual gyrus, right temporal lobe, left superior temporal gyrus, and left angular gyrus.

**Figure 8:**
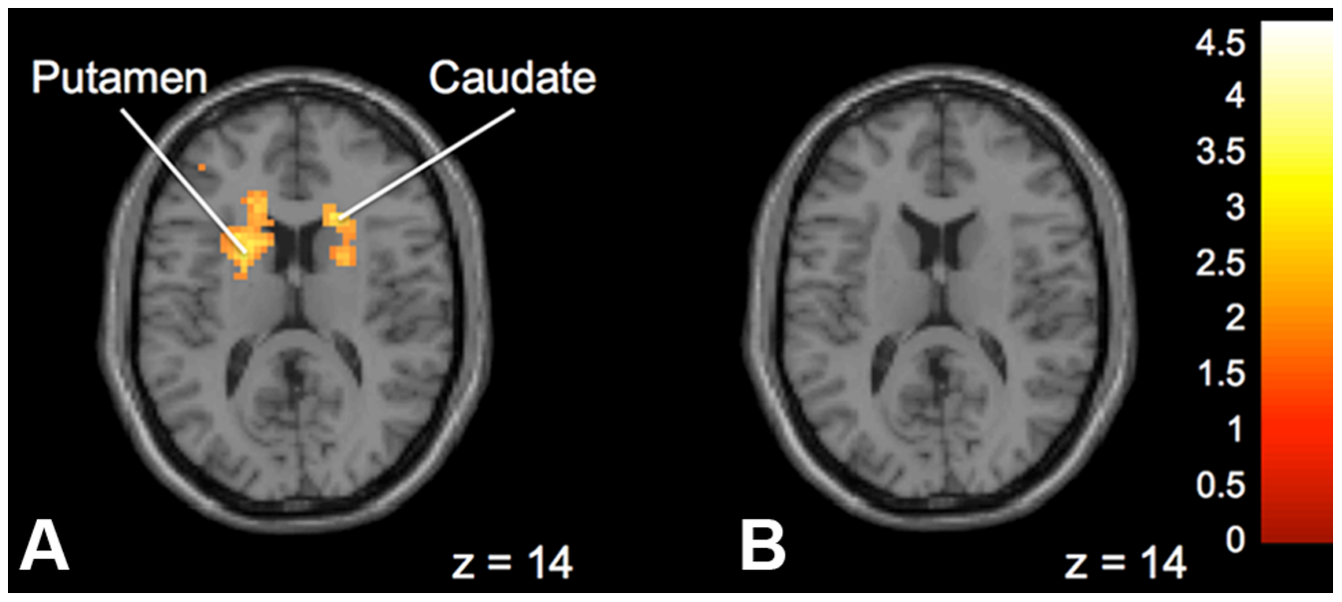
Brain activation for statistical contrast maps *NO ADVICE – ADVICE*. **a:** “Expert” group. No Advice trials elicited activation in the right caudate and left putamen for the “Expert” group. **b:** “Peer” group. No significant activation was found during No Advice trials for the “Peer” group. “z” coordinate provided at bottom right corners in MNI space. All results voxelwise statistical threshold at *t* = 2.500 (*p*<0.01) and a cluster threshold level of *p*<0.05 corrected for multiple comparisons.

In contrast, when comparing activation between groups for No Advice vs. Advice trials, significantly greater activation was found in the “Expert” group than the “Peer” group in the left posterior cingulate, bilateral thalamus, left insula, right caudate, right cingulate gyrus, bilateral medial frontal gyrus, left middle frontal gyrus and left frontal lobe white matter (Figure 7b). On within group tests, there was significant activation in the right caudate, right insula, left putamen, and frontal lobe white matter during No Advice trials as compared to Advice trials in the “Expert” group (Figure 8).

### Good Advice versus Bad Advice

There were no significant differences between the “Expert” and “Peer” groups when comparing Good advice with Bad Advice. When combining the groups we found significant differences between Good Advice and Bad Advice, with Good Advice trials eliciting significantly greater activation in the superior temporal gyrus, hippocampus and middle temporal gyrus compared to Bad Advice trials.

### Obedience over time

While no significant main effects were found for both Group and Run, a significant interaction effect did emerge in the dorsal anterior cingulate gyrus. Further analysis revealed that the “Expert” group showed significant deactivation relative to baseline compared to the “Peer” group in this area during the last two runs. This difference was driven by the “Expert” group demonstrating significant deactivation in this area in the last two runs compared to the first two runs; the “Peer” group did not show significant differences in this region across time.

## Discussion

The primary outcome of this study was to examine the cognitive processes underlying obedience and disobedience to “Experts” in financial decision-making. We found that there was greater activation in the anterior cingulate cortex (ACC) when choosing to disobey an “Expert” rather than a “Peer”. These findings for brain changes occurring when a financial “Expert” is disobeyed are consistent with previous research, which has linked changes in the ACC to error detection [27], [28]. It has also been suggested that one of the primary functions of the ACC is to monitor conflict [29], [30], and then help select an appropriate response [31]. Participants in the “Expert” group are required to consider both the advice of a (supposed) financial “Expert” as well as their own opinions before choosing to buy or not to buy each stock. When choosing to disobey the “Expert”, this conflict and integration process may take additional resources compared to choosing to disobey a “Peer”. Consistent with this are findings that right superior frontal gyrus activation has been linked to certainty-related processing [32], and in the present study participants in the “Expert” group may have required more certainty that disobeying the advice was the rational decision to make prior to choosing that action.

Because we employ a large cluster size threshold (*k* = 201), our tests apply an extremely conservative correction for multiple comparisons in all our analyses. This increases the power of our tests, making our findings less susceptible to Type II Errors than are most studies of this sort. Uniquely, the ACC and right superior frontal gyrus activation appears to differentiate between advice from an “Expert” vs. “Peer”. This suggests that the influence of advice on financial decision-making has more complex neurobiological links than previously recognized. These results support the hypothesis that there exists an obedience reflex to “Expert” advice that can be disengaged with the occurrence of some conflict processing. These findings would suggest that additional cognitive resources are required in order to switch off reflexive obedience, and oppose what an authority figure is recommending. However, when it comes to “Peer” advice, no reflex is present, thus no conflict processing occurs when the advice is disregarded.

In addition to activation in the ACC, there was also activation in the frontopolar cortex at the within groups level in the “Expert” group. In keeping with this finding it has been suggested that this region is important in the integration of multiple cognitive processes when pursuing a single higher behavioral goal [33]. Furthermore, both the pons and cerebellum are connected via subcortical projections to these prefrontal cortical areas, and have roles in decision-making, learning, working memory as well as the modulation of prefrontal function [34], [35], [36], and again this may explain why these regions are also activated during the investment task. That this activation occurred even though participants in the “Expert” group subjectively cited the same level of use of the advice as the “Peer” group suggests that the conflict of disobedience and the resolution of this conflict resulting in a disobedient financial decision occur subconsciously.

Previous research has implicated the anterior insula in nonconformity with an expert [15]; however, this activation appears to extend to nonconformity to a peer as no significant differences in activation in this area was found between the two groups. Once again, these results demonstrate that advice modulates activity in the brain in a more complex manner than previously supposed. Based on our results, it appears likely that some, but not all, brain regions affected by advice are differentially affected by whether advice is provided by either an “Expert” or a “Peer”. This finding adds to previous research on brain changes occurring during decision-making, and may need to be considered in future studies.

In addition to the primary findings, we also showed support for our first hypothesis that during the investment task, there was activation when comparing “Buy” vs. “Not Buy” decisions. When participants decided to “Buy”, they were taking a risk as the outcome could result in either monetary gain or loss. When participants chose to “Not Buy”, they were not risking the loss of any funds, and thus this could also be defined as a ‘risk-averse’ trial. Our findings are compatible with the previous literature in that during “Buy” decisions (i.e. risk-seeking choices) there was caudate activation, a region that has previously been linked in other studies to higher risk choices [22]. Conversely, in the “Not Buy”, or risk-averse choices, we found insula activation, a finding which has occurred in other studies during risk-avoidance choices [13], [22]. The insula is believed to be involved in interoceptive awareness (i.e. awareness of internal bodily senses) [37]. Thus, activation in the insula may indicate the possibility of an aversive outcome, such as punishment, and may lead a participant not to choose the more risky option [13], [37], in this case a “Buy” decision in our task. In addition, when comparing “Buy” to “Not Buy” we found that there was increased activation in the pallidum, a region which has previously been shown to precede advantageous actions [38]. Thus, our findings regarding individual decisions for “Buy” and “Not Buy” are compatible with the previous literature, validating our task.

When comparing “No Advice” to “Advice” trials. We found significantly greater activation in many areas in the “Expert” group compared the “Peer” group. These include frontal lobe, thalamus and left posterior cingulate. We suggest that it is possible in the “Expert” group that little cognitive effort was required when advice was presented, because of an obedience reflex to “Expert” advice, regardless of whether or not the advice was good or bad. In contrast, there was additional cognitive effort expended when no advice was given. Since the data suggests that the “Peer” group generally discounted the advice, they would therefore be expending relatively similar cognitive effort on both the “No Advice” and “Advice” trials, and therefore would not demonstrate any changes between these two activities.

The posterior cingulate has been implicated in the retrieval of episodic memory [39], semantic information [40] as well as self-reflective thought [41] indicating that part of this increased cognitive effort stems from switching from a reliance on the advice to relying on previous trials or previous experience to guide decision-making. Our findings are also compatible with previous research in which a lack of advice increased activation in the posterior cingulate cortex, inferior frontal gyrus and middle temporal gyrus [15] and advice modulates activity in the ventromedial pre-frontal cortex [20]. Consistent with previous research on decision-making in risk related tasks, the thalamus has also been implicated [42]. In the “Peer” group we found support for our hypothesis that this group would discount the advice, and thus they exerted greater cognitive effort (and more activation in these areas) in “Advice” trials than did the “Expert” group. In this scenario, it is suggested that the cost of gathering and evaluating the information provided on each stock is not seen to outweigh the risk of taking a cognitive shortcut and following the advice of a “Peer”, and this results in increased cognitive effort reflected in the brain changes detected by fMRI.

This hypothesis regarding our neuroimaging result was reflected in the behavior of our participants. Advice from the “Peer” was discounted, as was demonstrated by the significant decrease in obedient decisions compared to the “Expert” group (Figure 2). This follows previous fMRI research that participants value expert more than novice advice [11]. Participants in the “Peer” group also did not engage in their error detection and conflict monitoring mechanisms when disregarding the advice. In fact, when participants in the “Peer” group followed the advice presented there was greater activation in the hippocampus, insula and caudate. This may indicate that rather than ‘following’ the advice presented, these participants were making a decision that happened to agree with the advice based on risk assessment and previous trials. Furthermore, no significant activation was found during “No Advice” trials at the within groups level, indicating that the absence of advice may not have produced a similar increase in cognitive effort to that was found in the “Expert” group. Rather when the experimenter’s advice was presented, regions involved in semantic processing [40] and adjustments made to optimize performance [43] were activated, suggesting more cognitive effort despite the presentation of advice. What is particularly interesting in the present study is that even though the “Expert” was not present, or ever seen, the importance given to advice from this source had a meaningful impact on brain activation and behavior. This finding would support suggestions that even remote authority figures can have profound unconscious effects on financial (and perhaps other) behavior, and may in part explain how financial decisions can be significantly influenced by the current “understanding” or “knowledge” as interpreted by intermediaries [44], [45], [46], [47].

That there were no significant differences between the “Expert” and “Peer” groups when comparing “Good Advice” and “Bad Advice” indicates that the two groups did not differ in how they differentiated between the good and bad advice. “Good Advice” trials elicited significant activation in the superior temporal gyrus, hippocampus and middle temporal gyrus in both groups. This could indicate that participants were able to differentiate between good and bad advice and were learning and engaging in the “Good Advice” trials compared to “Bad Advice” trials. Nonetheless, it is important to note that all Good Advice trials occurred at the onset of the task and all Bad Advice trials occurred at the end of the task, and it is therefore conceivable that participants were generally more actively concerned about understanding the advice at the onset of the task compared to the end, and that this is reflected in the pattern of brain activation seen.

The interaction between obedience over time and advice lends more support for the role of the anterior cingulate cortex in decision-making as well as obedience. Specifically, the dorsal region of the anterior cingulate gyrus is associated with rational thought process and reward-based decision-making [48]. At the end of the task, the “Expert” group showed a significant decrease in activation in this region of the ACC compared to when the task first began when obeying the presented advice; however, the “Peer” group did not show this change in activation across time. Decreased activation in the ACC has been found in depressed patients in decision-making/reward anticipation and is believed to reflect a lack of awareness or concern for outcomes [49]. Based on these findings coupled with the “Expert” groups greater behavioral obedience compared to the “Peer” group in the final runs, we hypothesize that over time, advice from a seemingly trusted source, such as an expert, may lead to a similar reduced awareness in decision-making as seen in depressed patients. When a peer, with no social context of being particularly trustworthy, provides the advice that advice does not elicit the same pattern of activation, allowing the individual to make more rational decisions over time. In the case of our task, more rational decision-making led to less obedient decisions later in the task, as demonstrated by our “Peer” group.

Our hypotheses regarding utility of trust and the positive or negative emotions associated with obeying or disobeying the advice were not supported. It is conceivable that our task did not elicit strong enough emotional reactions from participants to reveal significant activation at our high threshold.

It is important to recognize that this research may illuminate only one aspect of financial decision-making. Personality factors can also be important, for example, one study suggested that those students who have a higher risk taking and more positive attitude to gambling may be more likely to pursue careers in the financial industry [50]. The present study was not a double-blind study, so it is possible that some biases may have existed. Furthermore, most of the individuals taking part were university students and it may not be appropriate to generalize these findings to the general population. While our control was a good one that served the purpose of our study, it should be noted that a true control would have involved an additional scenario where participants were told that the advice was random. Nonetheless, given the size of the study, the robustness of the findings about brain activation when individuals defy the advice of an alleged financial “Expert”, and that the findings are compatible with the existing literature, we believe these findings add meaningfully to the existing understanding of why individuals make irrational financial decisions, particularly when under the influence of advice.

## Conclusions

In conclusion, the present results suggest that when individuals defy authority, or believe they are doing this (in this case by disobeying an “Expert”) to make an investment decision there is increased activation of the ACC and superior frontal gyrus, and that such activation is in part responsible for disobedience. These results specify the differences in activation to the level of source and benefit of advice. Increased awareness of this may allow strategies to be developed to help both individuals and groups avoid inappropriate financial decisions. It is also possible that these results may have wider implications about the brain mechanisms underlying obedience. When the advice is sought from someone deemed to be an expert, it is conceivable that this influence can have negative outcomes for individuals, as they might offload cognitively and defer to the expert without forming independent judgments,.

In summary, in this novel study the major findings are firstly that they support suggestions of an obedience reflex to “Expert” advice, and secondly that it is conceivable that the mechanism by which this acts might involve cognitive offloading which occurs when “Expert” advice is present. These processes may, in part, explain some of the reasons why individuals choose to follow the advice of “Experts”, financial and otherwise.
